# Diurnal Variation of Plasma Extracellular Vesicle Is Disrupted in People Living with HIV

**DOI:** 10.3390/pathogens10050518

**Published:** 2021-04-24

**Authors:** Wilfried Wenceslas Bazié, Benjamin Goyer, Julien Boucher, Yuwei Zhang, Delphine Planas, Debashree Chatterjee, Jean-Pierre Routy, Michel Alary, Petronela Ancuta, Caroline Gilbert

**Affiliations:** 1 Axe de Recherche Maladies Infectieuses et Immunitaires, CHUL, Centre de Recherche du CHU de Québec-Université Laval, Québec, QC G1V 4G2, Canada; benjamin.goyer@crchudequebec.ulaval.ca (B.G.); julien.boucher.2@ulaval.ca (J.B.); 2 Programme de Recherche sur les Maladies Infectieuses, Centre Muraz, Institut National de Santé Publique, Bobo-Dioulasso 01 BP 390, Burkina Faso; 3 Département de Microbiologie, Infectiologie et Immunologie, Faculté de Médecine, Université de Montréal, Montréal, QC H3T 1J4, Canada; zhangyw927@gmail.com (Y.Z.); delphine.planas@pasteur.fr (D.P.); debashree.chatterjee@umontreal.ca (D.C.); petronela.ancuta@umontreal.ca (P.A.); 4 Centre de Recherche du Centre Hospitalier de l’Université de Montréal, Montréal, QC H2X 0A9, Canada; 5 Chronic Viral Illness Service and Division of Hematology, McGill University Health Centre, Montréal, QC H4A 3J1, Canada; jean-pierre.routy@mcgill.ca; 6 Infectious Diseases and Immunity in Global Health Program, Research Institute, McGill University Health Centre, Montreal, QC H4A 3J1, Canada; 7 Axe de Recherche Santé des Populations et Pratiques Optimales en Santé, Centre de Recherche du CHU de Québec-Université Laval, Québec, QC G1S 4L8, Canada; michel.alary@crchudequebec.ulaval.ca; 8 Département de Médecine Sociale et Préventive, Faculté de Médecine, Université de Laval, Québec, QC G1V 0A6, Canada; 9 Institut National de Santé Publique du Québec, Québec, QC G1V 5B3, Canada; 10 Département de Microbiologie-Infectiologie et d’Immunologie, Faculté de Médecine, Université Laval, Québec, QC G1V 0A6, Canada

**Keywords:** extracellular vesicles, HIV−1, microRNA, miR-29a, miR-29b, miR-92, miR-155, miR-223, circadian clock

## Abstract

Background: Several types of extracellular vesicles (EVs) secreted by various immune and non-immune cells are present in the human plasma. We previously demonstrated that EV abundance and microRNA content change in pathological conditions, such as HIV infection. Here, we investigated daily variations of large and small EVs, in terms of abundance and microRNA contents in people living with HIV (PLWH) receiving antiretroviral therapy (HIV+ART) and uninfected controls (HIV−). Methods: Venous blood samples from n = 10 HIV+ART and n = 10 HIV− participants were collected at 10:00 and 22:00 the same day. Large and small plasma EVs were purified, counted, and the mature miRNAs miR-29a, miR-29b, miR-92, miR-155, and miR-223 copies were measured by RT-PCR. Results: Large EVs were significantly bigger in the plasma collected at 10:00 versus 22:00 in both groups. There was a significant day–night increase in the quantity of 5 miRNAs in HIV− large EVs. In HIV+ART, only miR-155 daily variation has been observed in large EVs. Finally, EV-miRNA content permits to distinguish HIV− to HIV+ART in multivariate analysis. Conclusion: These results point that plasma EV amount and microRNA contents are under daily variation in HIV− people. This new dynamic measure is disrupted in PLWH despite viral-suppressive ART. This study highlights a significant difference concerning EV abundance and their content measured at 22:00 between both groups. Therefore, the time of blood collection must be considered in the future for the EV as biomarkers.

## 1. Introduction

During the last decade, extracellular vesicles (EVs) have been identified in many biological fluids and have gained importance as cell-to-cell communicators [1,2]. Based on their cellular origin and/or biological function or based on their biogenesis, EVs are classified as exosomes, microvesicles, and apoptotic bodies [3,4,5,6]. EV harbor specific subsets of bioactive cargos representing their originating cell types and pathophysiological conditions [4,7]. Importantly, attention has been focused on EV as non-invasive biomarkers for the diagnosis and prognosis of diseases [8,9,10,11,12]. Moreover, emerging roles for therapeutic opportunities regardless of multifaceted biological functions are observed [9]. They have been reported to carry cellular components that have functional effects on neighboring or distant cells, including mRNA and microRNA, other noncoding RNA, cytoplasmic and membrane proteins, and lipids [7].

The microRNAs (miRNAs) are a class of short RNAs that play a crucial role in almost all biological pathways by regulating post-transcriptional silencing of target genes [13,14,15]. The miRNAs produced by cells can be transported from donor cells to recipient cells by EV, lipoproteins, and ribonucleoproteins [16,17,18]. The miRNAs in the EV are stable in biological fluids since they are protected by a double lipid layer [19,20]. The presence of miRNAs within EV isolated from circulating body fluids has stimulated many studies aiming to investigate EV diagnostic and prognostic potential as the fact that vesicular miRNA content is affected by the pathological state of the cells or tissues [21,22].

Based on their implication on the physiological process, some studies have described temporal variation of EV in circulating blood. The amount of EV in blood at a given time results from a delicate balance between production and uptake of EV by various tissues and organs and could be influenced by multiple factors, including exercise, time since last meal, gender, age, and circadian rhythm [23,24].

Circadian rhythms are approximately 24 h exhibited by most organisms, from unicellular to vertebrates [25]. Circadian variations drive physiological and cellular adaptations in various processes and regulate or optimizes the function of cells, organs, systems, and behavior throughout the 24 h duration of a day [26]. Many physiological and behavioral processes, including hormone secretion and regulation, metabolism, immune responses, and sleep-wake cycles, are modulated by the circadian clock [26,27]. Circadian variations of the immune response, from the trafficking of immune cells to the activation of innate and adaptive immunity, to host–pathogen interactions, have been described [28,29,30]. Cells of the innate immune system, such as neutrophils, macrophages, and monocytes, whose number changes dynamically over the day, exhibit circadian patterns of migration from the blood to tissues [28,29,31,32,33]. Furthermore, the diurnal variation in T cell proliferation and distribution modulate T cell interaction with antigen-presenting cells and influence immune responses to antigens and bacterial infections [30,34,35]. In addition, circadian clocks impact the nature and amplitude of inflammatory responses induced by pathogens [29,30]. Some pathogens are described to induce a circadian disruption, thus promoting their expression and may contribute to pathogenesis. Yang et al., reported that hepatitis B virus encoded X (HBx) perturbed several clock genes and transcriptional levels with increased transcription of Clock, Per1, and Per2 and significantly lower levels of Per1-3 and Cry2 mRNA [36]. Human Immunodeficiency Virus (HIV) and Simian Immunodeficiency Virus infection have been associated with the disruption of circadian-regulated physiological processes [37], a disturbance of the circadian rhythm of T and B lymphocytes [38,39], and a resetting of the circadian clock [40].

Given the impact of diurnal variations on numerous blood-borne factors and the increasing interest in circulating blood EV and miRNAs as new biomarkers for the pathological state, it is necessary to elucidate their physiological nychthemeral variations. Some studies reported diurnal variations of circulating extracellular vesicles and miRNAs in human plasma. Danielson et al., observed variations in the relative size and concentration of EVs in healthy adult plasma across the course of a day and suggesting that there are diurnal variations in the number and size distribution of circulating EV populations [23]. Investigating a panel of 92 miRNAs in plasma samples from young male healthy volunteers repeatedly sampled nine times during 24 h, Heegaard et al., demonstrate that a third of the measurable plasma miRNAs exhibit a rhythmic behavior and are distributed in two main phase patterns [41].

Despite the circadian variation of EV size and concentration and cell-free miRNAs in plasma, the day-to-night variation of EV miRNA content has not been investigated mainly in the state of HIV infection. It is now well known that EV and microRNAs are essential players in HIV infection immunopathogenesis [42,43,44,45,46], and the potential of these molecules and EV as biomarkers for HIV infection management has recently emerged [47,48]. Based on their dimension, EVs are classified as small and large vesicle [6] and all subtypes must be checked and taken account for biomarkers analysis.

In this manuscript, we investigated the daily variation of a panel of five miRNAs inside large and small EVs purified from HIV− and PLWH participants plasma. We aimed to describe the daily variations of EV size, abundance, and microRNA contents and how HIV infection impacts this variation. We focused on miRNAs previously reported to play an important role in HIV physiopathological mechanisms, such as miR-29a and b, miR-92, miR-155, and miR-223 [49].

## 2. Results

### 2.1. Days versus Night EV Abundance Variation Was Disrupting in PLWH

**Figure 1:**
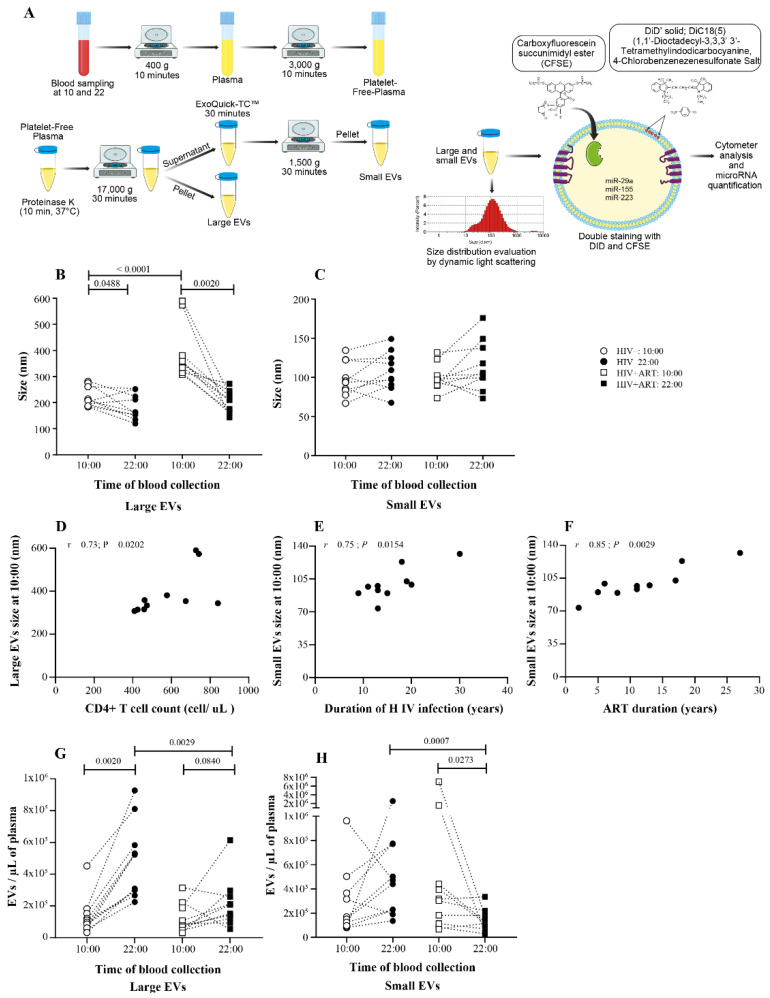
Daily variations in the size and abundance of plasma large and small EVs in HIV+ART and HIV− study participants. Large and small EVs were purified from proteinase K pretreated platelets-free plasma of uninfected (HIV−, n = 10) and HIV infected ART-treated subjects (HIV+ART n = 10) sampled at 10:00 and 22:00 the same day as illustrated in panel (**A**). The Size distribution of large (**B**) and small (**C**) EVs was evaluated using dynamic ligth scatering. Large EVs size at 10:00 correlation with CD4 T cell count in HIV+ART (**D**), small EV size at 10:00 correlation with HIV infection duration (**E**,**F**) with ART duration. Then, purified EVs were stained with lipophilic fluorescent tracer dye DiD and carboxyfluoresceine diacetate succinimidyl ester (CFSE) to count total vesicles (**A**). Large (**G**) and small (**H**) EVs abundance variation between 10:00 and 22:00 in HIV− and HIV+ART participants. Wilcoxon matched-pairs signed-rank test, Mann–Whitney test and Spearman r correlation test were used for statistical analysis.

To evaluate daily variation in EV abundance for both type of EVs (large and small), blood from HIV+ART and HIV− study participants were sampling at 10:00 and 22:00 the same day. Large and small EVs were purified from proteinase K pretreated plasma, and their size was evaluated by dynamic light scattering (DLS) (Figure 1A). We observed that size of large EVs from HIV+ART (387.6 ± 104.9 nm at 10:00 versus 204.7 ± 41.08 nm at 22:00; mean ± standard deviation) as well as uninfected (220.4 ± 38.27 at 10:00 vs. 182.1 ± 48.63 nm at 22:00), were significantly increased at 10:00 compared to 22:00 for both group of participants (Figure 1B). In contrast, small EVs in both groups ((HIV+ART: 10:00 = 99.75 ± 16.75 vs. 22:00 = 114.5 ± 31.52 nm) and (uninfected: 10:00 = 98.02 ± 21.89 vs. 22:00 = 107.6 ± 24.63 nm)), show nonsignificant difference (Figure 1C). Large EVs size at 10:00 was significantly higher in HIV+ART but no difference was seen at 22:00 and in small EVs (Figure 1B,C). As we have previously shown, large EVs size at 10:00 was positively correlated with CD4 T cell count in HIV+ART subjects (r = 0.73, *p* = 0.0202; Figure 1E) [47]. Small EVs size at 10:00 was respectively correlated with HIV infection duration (r = 0.75, *p* = 0.0154; Figure 1F) and ART duration (r = 0.85, *p* = 0.0029; Figure 1G). All together, these results show that only large EV was subject to circadian variation in both groups and HIV infection does not disturb this physiological state. Moreover, there is an interdependence between small EVs size at 10:00 and HIV infection or ART duration.

Knowing that EV size varied between a.m. and p.m. as well as cells numbers [28,29,31,32,33], we then evaluated plasma EV abundance variation during the nychthemeral cycle. Large and small EVs purified from plasma sampled at 10:00 and 22:00 the same day were stained with DID a lipophilic fluorescent tracer dye for EV lipidic bilayer and carboxyfluoresceine diacetate succinimidyl ester (CFSE) a cell-permeable dye then analyzed in a cytofluorometer (Figure 1A). Results showed that large EVs were significantly more abundant at 22:00 in HIV− and this variation between 10:00 and 22:00 was lost in HIV+ART subjects (Figure 1G). Regarding small EVs, no difference was seen in uninfected subjects and in contrast to HIV+ART subjects where EVs were more abundant at 10:00 than 22:00 (Figure 1H). Relatively to HIV+ART, large EVs (*p* = 0.0029) and small EVs (*p* = 0.0007) were significantly more abundant in HIV− at 22:00 (Figure 1G,H). Unexpectedly, no correlation was found between HIV+ART EVs abundance and CD4, CD8 T cell count, or CD4/CD8 ratio. Taken together, these results show that plasma EV abundance is dynamic during the day/night cycle and that HIV infection disturbs large and small EV abundance diurnal variations.

### 2.2. Significant Daily Variation of microRNA in Large EV in Control Subject

**Figure 2:**
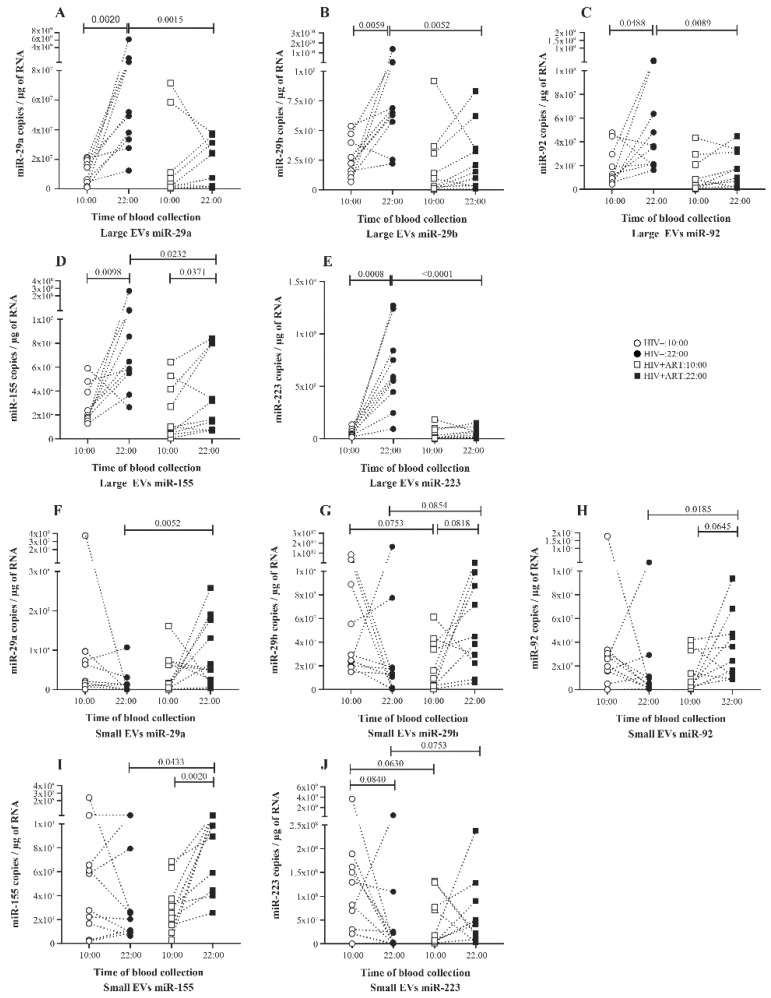
Daily variations of microRNA expression in plasma large and small EVs of HIV− and HIV+ART study participants. Total RNA was extracted from large and small EVs purified from proteinase K pretreated platelets-free plasma of uninfected (HIV−, n = 10) and HIV infected ART-treated subjects (HIV+ART, n = 10) sampled at 10:00 and 22:00 the same day. Matures miRNAs were reverse transcribed and quantified as described in the Materials and Methods section. Large EVs (**A**–**E**) and small EVs (**F**–**J**) miRNA expression level at 10:00 and 22:00 were presented. Wilcoxon matched-pairs signed rank test and Mann–Whitney test were used for statistical analysis.

Investigating day to night variations of a panel of five miRNAs in plasma EV, 10 HIV uninfected patients were sampled at 10:00 and 22:00. Total RNA was extracted from purified plasma EV, and mature EV-miRNAs were quantified by RT-qPCR. The analysis shows a significant variation with an increase in the quantity of all five miRNAs miR-29a (Figure 2A), miR-29b (Figure 2B), miR-92 (Figure 2C), miR-155 (Figure 2D), and miR-223 (Figure 2E) in large EV of the 22:00 samples compared to the 10:00 ones. In small EVs, no significant variation was seen for five miRNAs quantities (Figure 2F–J).

**Figure 3:**
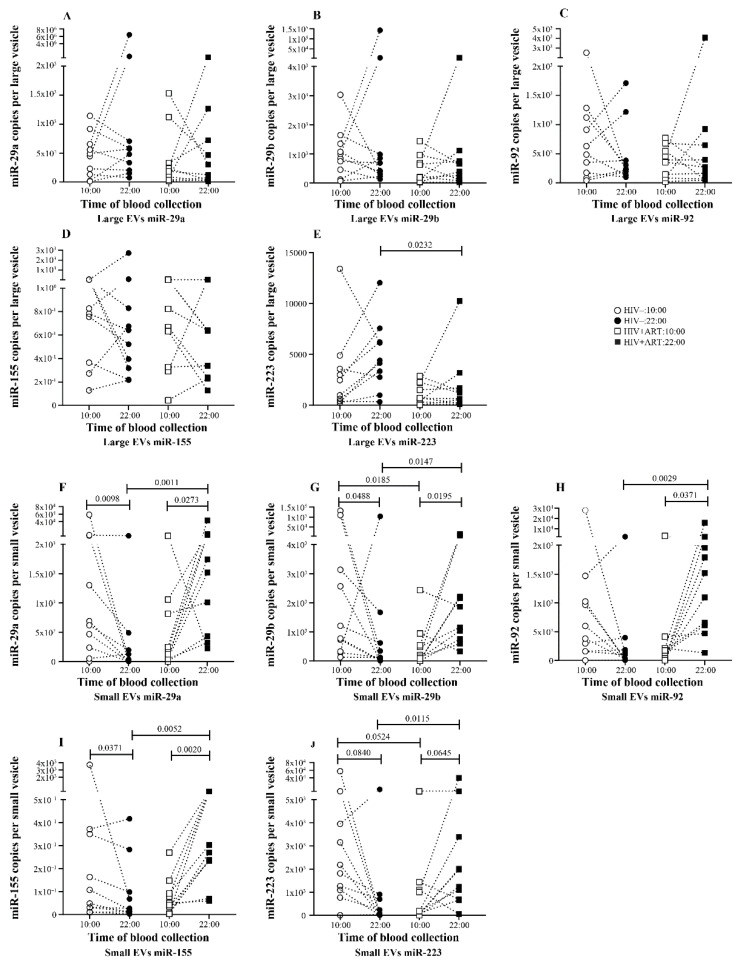
Daily variations of microRNA level expression as copies per large and small vesicle of HIV− and HIV+ART study participants. Copy number of each miRNA molecule per large and small vesicle is expressed for HIV− and HIV+ART study participants large EVs (**A**–**E**) and small EVs (**F**–**J**) at 10:00 and 22:00. Wilcoxon matched-pairs signed rank test and Mann–Whitney test were used for statistical analysis.

To better understand EV miRNA day to night variation, EV-miRNA quantity was expressed as copies per vesicle as described in the methods section. Unlike the total amount in large EVs, no variation was seen in five miRNAs expression per vesicle between 10:00 and 22:00 (Figure 3A–E). In small EVs, we observed a trend to have more miRNA per vesicle at 10:00 than 22:00 and this variation was significant for 3 of 5 miRNA (Figure 3F,G).

Regarding the miRNA expression in large EVs compared to small EVs, we observed a globally opposite tendency to have more miRNA in the small EVs at 10:00 and in large at 22:00 (Figure S1A,C,E,I). This variation is significant for miR-29b at 10:00 samples and for miR-29a, miR-223 at 22:00 samples. The miR-155 is identified as being the only one characterized by enrichment in the large EV in 10:00 and 22:00 (Figure S1G). Together, these findings suggest a physiological variation of the miRNA quantities in EV during the day/night cycle particularly in large EVs as observed with larger EVs abundance. Moreover, these results denote preferential enrichment of EV subtype in miRNA depending on the time of the day.

### 2.3. Increasing Amount of miR-155 in Large and Small EV at Night in PLWH ART-Treated

We next investigated what happened in EV-miRNA day-night variation in PLWH. Ten ART-treated PLWH with undetectable HIV viral load were sampled at 10:00 and 22:00, EVs were purified from platelet-free plasma, and miRNAs were quantified as carried out for the control subjects. In large EVs, compared to the 10:00 sample, only miR-155 quantity increases significantly in the 22:00 sample (Figure 2D). As shown in Figure 2A–C, and E, no variation was seen in the amount of miR-29a, miR-29b, and miR-223 between the 10:00 and 22:00 samples. In small EVs, the trend to have more miRNA at 22:00 was significant for miR-29b, miR-92, and miR-155 (Figure 2F–I). Concerning miRNA expression level per large EVs, no variation was observed between the 10:00 and 22:00 samples for all five miRNAs (Figure 3A–E). In contrast, small EVs miRNA level per vesicle was significantly more abundant in 22:00 sample for miR-29a, miR-29ab, miR-92, and miR-155 (Figure 3F–I). Comparing miRNA quantity in small and large EVs, as observed in control patients, miR-155 was also more enriched in large EVs at 10:00 and 22:00. The amount of miR-29a and miR-29b were enriched in small vesicles at 22:00 (Figure S1B,D).

### 2.4. Disturbance of the Circadian Pattern of EV-miRNA Content in PLWH ART-Treated

By comparing EV-miRNA expression patterns in HIV+ART and HIV uninfected, we observed that in large EVs the miRNA variation between 10:00 and 22:00 was lost in HIV+ART excepted for miR-155 (Figure 2A–E). Regarding miRNA quantity at 10:00 and 22:00, miR-29a (Figure 2A), miR-29b (Figure 2B), miR92 (Figure 2C), miR-155 (Figure 2D), and miR-223 (Figure 2A) were significantly more abundant in HIV− large EVs compared to HIV+ART at 22:00. Likewise, only miR-223 level per large vesicle was higher in HIV− at the same time. About small EVs miRNA quantity, an opposite trend was observed with more miRNA in HIV+ART small EVs at 22:00. This trend was significant for miR-29a (Figure 2F), miR-92 (Figure 2H), and miR-155 (Figure 2I). Concerning miRNA expression per small vesicle, all five miRNAs were significantly higher in HIV+ART compared to HIV− at 22:00. Surprisingly, no significant difference was seen in large and small EVs miRNA quantity between HIV− and HIV+ART at 10:00 (Figure 2A–J).

**Figure 4:**
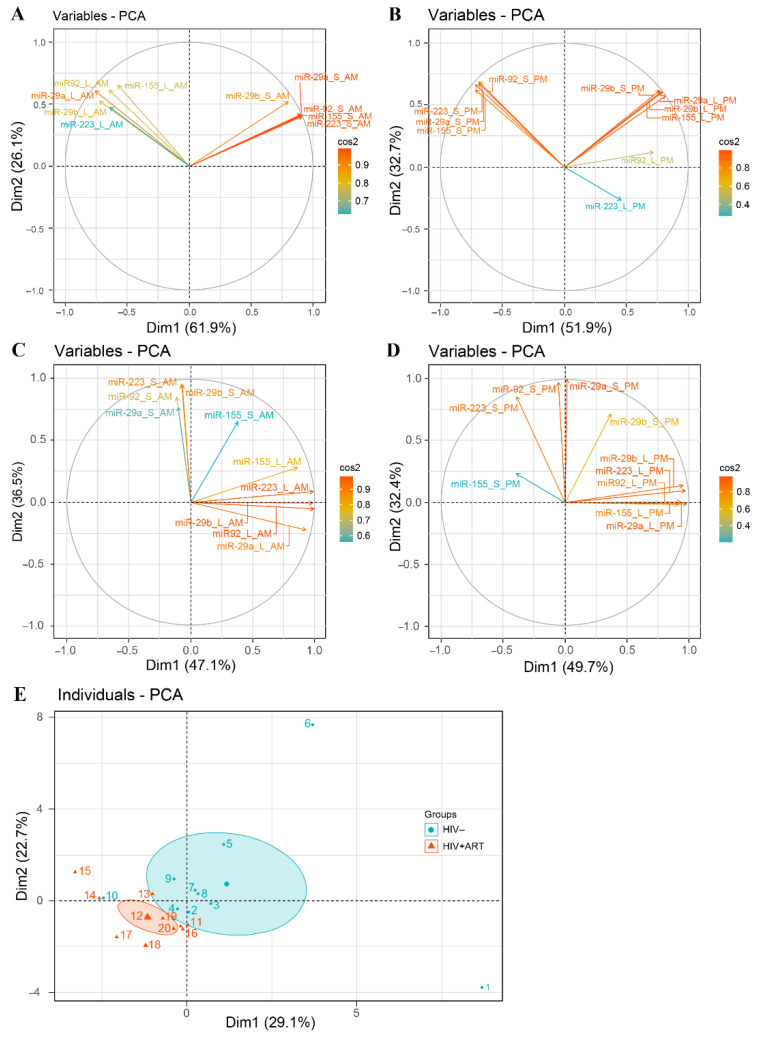
Principal component analysis of daily variations in EV mRNA expression in HIV+ART and HIV− study participants. Correlation graph of large and small EVs miR-29a, miR-29b, miR-92, miR-155, and miR-223 content at 10:00 and 22:00: (**A**,**B**) present graph results for HIV− and (**C**,**D**) graph for HIV+ART. Correlation graph of 10 control subjects (individuals 1–10) and 10 HIV infected subjects (individuals 11–20) according to their large and small EVs miRNA content at 10:00 and 22:00.

Considering the quantity of all five miRNAs in multivariate analysis, we noted a different relationship profile between variables at 10:00 and 22:00 in HIV− (Figure 4A,B) and HIV+ART (Figure 4C,D). In HIV−, we observed a strong positive correlation between the quantity of different miRNAs in large and small EVs at 10:00 and 22:00. Moreover, we observed a negative correlation between the quantities of miRNAs in large and small at a.m. and p.m. In HIV+ART, there is a weak correlation between the quantity of these miRNAs in large and small both at 10:00 and 22:00. Moreover, considering together the expression of 5 miRNAs in large and small EVs at 10:00 and 22:00 in principal component analysis, HIV− and HIV+ART subjects are clustered for the most part (Figure 4E and Figure S2A,B). This analyzes permits the separation of HIV− from HIV+ART based on EV-miRNA content. These results highlight temporal oscillation of EVs miRNA contents patterns and suggest that HIV infection disrupts EV-miRNA day-night variation by inversing miRNA circadian pattern in small EVs. These results raise the question of sampling time for comparisons between HIV− infected and control subjects.

### 2.5. Small EVs miR-155 a Biomarker of Immune Activation in PLWH ART-Treated

In the relationship between EV-miRNA content and immune parameters used for clinical follow-up of PLWH such as CD4, CD8 T Cell, and CD4/CD8 ratio, a correlational analysis was performed for control and HIV+ART subjects (Table 1). The analysis showed that CD4/CD8 ratio correlated with the 5 miRNAs level in large EVs at a.m. This correlation was significant for miR-29b (r = 0.84, *p* = 0.0064) and miR-92 (r = 0.84, *p* = 0.0071) and was not seen among HIV+ART subjects. Some correlations between clinical parameters and miRNAs expression per vesicle have been observed. However, these correlations do not agree with those observed with the total amounts of miRNAs. Concerning age, it was inversely correlated with large EVs miR-29a copies per vesicle (r = −0.76, *p* = 0.0218) and positively with small EVs miR-29a copies per vesicle (r = 0.81, *p* = 0.0112) in 10:00 samples. The large (r = −0.76; *p* = 0.0218) and small (r = −0.76, *p* = 0.0143) EVs miR-92 copies per vesicle at 22:00 was inversely correlated with age. The miR-155 level per large vesicle (r = 0.74, *p* = 0.0267) and per small vesicle at 10:00 (r = −0.71, *p* = 0.0377) was respectively positively and inversely correlated with age. Regarding HIV+ART subjects, only small EV-miR-155 level at 22:00 (r = 0.75, *p* = 0.0255) was positively correlated with CD8 T cell count. Taken together, these results showed some relationships between EV-miRNA contents and clinical parameters in physiological conditions, and which are lost with immune disorders induced by HIV infection. The relationships between EV-miRNA content and age provided evidence of the role of miRNAs in senescence. Molecular studies of aging and miRNAs would provide a more comprehensive understanding. The relationships between vesicular miR-155 at 20:00 and CD8 T cell count strengthens data on the implication of miR-155 in immune activation and inflammation during HIV infection and, therefore, its implication in HIV infection immunopathological mechanism and perhaps a functional biomarker. Further, these observations highlight that in addition to miRNA total quantification, EV-miRNA expression per vesicle could also be a most remarkable biomarker.

## 3. Discussion

Previously, our team showed that EVs found in the plasma of ART-naive patients were larger and more broadly distributed than those from any other group of subjects, but no difference was seen between control and ART-treated patients with suppressed viral load [48]. In this study, we highlight a difference in plasma EV contain between successful ART-treated patient and uninfected control in regard of time sampling.

We observed an increasing trend in EV abundance at p.m. This trend was significant only for large EVs in control subjects and was not so among PLWH. Surprisingly, large and small EVs abundance was greater in control patients than in HIV+ART at p.m. This observation contrasts with our previous results in a.m. samples and a study that describes that HIV infection induces an increased number of circulating EV that originate from a broad population of persistently activated immune cells [50]. However, in the study that confirmed an increased production of EV during HIV infection, the sampling was done essentially during the day [50]. Physiologically, the number of the major leukocyte subsets in blood, including neutrophils, monocytes, CD4 T cell, CD8 T cell, NK cells, and eosinophils varies throughout the day, reflecting bone marrow output and emigration from the blood into tissues [51,52,53]. This spatiotemporal distribution of leukocyte subsets is driven by the circadian expression of pro-migratory molecules expressed on the leukocytes and the endothelium [52,53]. Systemic circadian signals and cell-intrinsic molecule clocks contribute to the oscillatory expression of these pro-migratory molecules [52]. Comparative evaluation of the expression level of these pro-migratory molecules in controls and PLWH could confirm our observations. Our finding could suggest that HIV infection causes disruption of the circadian clock system and are supported by results of other studies which reported that HIV alters circadian rhythms through the light entrainment pathway [40] with as consequence a disturbance of the circadian rhythm immune cells such as T and B lymphocytes [38,39].

We also showed that size of large EVs are greater at 10:00 than 22:00 for HIV− and HIV+ART-treated group. Contrary to our results, Danielson et al. [23] noticed that EV collected in the evening had the most extensive range of sizes than morning EV. Comparing to control, HIV+ART large EVs were larger at 10:00. In this study, no difference was observed between control and HIV+ART at any time regardless of small EVs size. Similarly, Chettimada et al. [54], reported no significant difference in EV size in control subjects, viremic and aviremic HIV−positive groups. The difference in reported results could be explained at least by three factors, including (1) samples are not always taken at the same time of day, (2) other factors not considered can influence EV size, (3) difference in EV purification methods such as ultracentrifugation or precipitation with or without proteinase K pretreatment for example. It should be noted that our method (Figure 1A) sequentially separates two subpopulations of different sizes EV from proteinase K pretreated plasma while the other studies focus either on exosomes or total plasma EV. For more comparability between studies, it would be interesting to harmonize the patient sampling periods and EV purification methods.

Our results show interdependence between small EVs size at 10:00 and HIV infection or ART duration. The size of small EVs increases with the duration of infection and ART treatment. Emerging evidence suggests direct links between autophagy and exosome biogenesis through shared molecular machinery and a subset of the autophagy machinery may contribute to exosome biogenesis [55,56]. During HIV infection, the antiviral and immune properties of autophagy are severely dysregulated through a plethora of direct and indirect mechanisms [57,58]. In particular, the HIV proteins TAT, NEF, and ENV are involved in this regulation by either blocking or stimulating autophagy through direct interaction with autophagy proteins and/or modulation of the mTOR pathway [59,60,61,62]. Likewise, ART drugs [63,64,65] and aging [66,67] have been associated with dysregulation of autophagy. These different factors associated with dysregulation of autophagy may contribute to autophagosome and multivesicular bodies fusion [68] and induce larger EV release.

Concerning EV miRNA content, knowing that cell-free miRNAs exist in the bloodstream incorporated into EVs [19] and associated with high-density lipoproteins [16] and Ago2 protein [18], we first evaluate the impact of proteinase K digestion step on EV miRNA quantification. As reported by over studies, the added of proteinase K step (Figure S3) significantly decreases the amount of miR-155 and miR-U6 in purified large EVs. This suggests that the proteinase K treatment is required for the efficient removal of extra-vesicular RNA and supports our EV purification method (Figure S4) in accord with MISEV2018 recommendation. In large EVs, all five miRNAs show an increase in the 22:00 samples and follow the same distribution as the abundance of vesicles between 10:00 and 22:00. Compared to controls, there is an abolition of the a.m.–p.m. variation in the amount of miR-29a, miR-29b, miR-92, and miR-223 in HIV+ART. In the controls’ small EVs, despite the tendency to have more EV at p.m., no significant difference was seen for miRNA between a.m. and p.m. In HIV+ART subjects, small EVs were more abundant at a.m. and contrast with miRNA quantity variation. In addition to showing an increase for miRNA in HIV+ART at p.m., this opposite variation between small EVs abundance and miRNA quantity suggests that some EVs are miRNA-free, and methods to identify and purify EV that contain miRNAs must be developed for better characterization of these EVs. Globally, we observed an inverse trend in large and small EVs miRNA content variation at a.m. and p.m. between control and HIV+ART. Evidence shows that the expressions of the mature or precursor forms of some miRNAs exhibit circadian and/or diurnal rhythms and are suggested to be involved in circadian rhythm regulation and physiological function [69,70]. The miRNAs miR-155, miR-27b-3p, miR-211, and miR-142 have been described to regulate the rhythmic expression of Bmal1 mRNA and protein levels [69,71,72]. HIV infection has been associated with irregularities in physiological functions related to circadian rhythm [37,73]. HIV *Tat* is known to directly affect the mammalian master circadian pacemaker located in the hypothalamic suprachiasmatic nucleus, which underlies lentiviral circadian rhythm dysfunction, and this effect of *Tat* occurs only during the subjective night [40,74]. Moreover, HIV infection was described to induce a change in the cellular miRNA profile and could explain observed variations [75,76].

Our results show a variation in miRNAs quantity according to EVs subpopulation, daytime, and HIV infection status. To our knowledge, few studies have been reported on the physiological rhythmicity of plasma miRNAs, and it is not well known if any of the cellular and extracellular miRNA exhibits diurnal variation. The time-of-day dependent distribution of 26/79 plasma miRNAs in healthy volunteers with some miRNAs peaking at night and some during the day have been reported by Heegaard et al. [41]. The miRNAs miR-155 and miR-223 were described to have a peak respectively at night and day [41]. This tendency to have a variation in the quantity of miRNA depending on the daytime and type of EV underlines the need to consider the different subpopulations of EV and the sampling time in the various explorations of their biomarker role. Further research will be needed to understand the mechanism of circadian generation and regulation of cellular and cell-free miRNA expression as well as cellular origin.

When we compare both groups, we realize that despite ART duration of more than ten years with an undetectable viral load among PLWH, the normalization of the CD4 T cell count contrasts with a higher number of CD8 T cells and a low CD4/CD8 ratio in PLWH. Data have shown that immune dysfunction and chronic inflammation are not completely resolved during ART despite suppression of viral replication and restoration of CD4 T cell count in peripheral blood [77]. These data highlight the need for new and more functional biomarkers to manage immune activation that increases the risk of non-AIDS-linked morbidities, including cancer and cardiovascular diseases, metabolic syndrome in PLWH under ART [77,78]. Exploring the landscape of immune cell components in EVs, particularly T cell fraction that play a key role in HIV infection we observed that large and small EVs bearing CD8 marker were more abundant than CD4 vesicles (Figure S5). This preponderance of CD8 vesicles associated with the persistence of a high number of CD8 despite the ART interpellates us on the role of these CD8 vesicles in the persistence of immune activation in PLWH despite ART and undetectable viral load. This could help us to fully understand the immune activation in PLWH for better management.

The microRNA miR-155 is the only miRNA that shows the same direction of variation between 10:00 and 22:00 in large and small EVs of HIV+ART. Moreover, it was also the only miRNA that showed a correlation with the count of CD8 T cells a stigma of immune activation. In PLWH, Jin et al. [79] showed the same correlation between the miR-155 level and CD8 T cell count as observed in our study. They stipulate that miR-155 is a biomarker of T-cell activation and immune dysfunction in HIV−1-infected patients [79]. Effectively, miR-155 has been documented to enhance CD8 T cell expansion during acute viral infection and cancer [80,81,82]. During chronic infection, miR-155 was described to be a key regulator of T cell responses by fostering the development, accumulation, and long-term durability of a large population of terminally differentiated PD-1 T cells [82,83]. Overexpression of miR-155 in virus-specific CD8 T cells enhanced expansion and long-term persistence during chronic viral infection [83]. In HIV infection, expression of miR-155 is increased and higher expression correlates with the increased disease [84]. Likewise, miR-155 was described to participate in the regulation of the biological clock. Curtis et al., identified that miR-155 controls Bmal1 mRNA and protein levels in myeloid cells, and leading to alterations in clock function and circadian control of inflammation [69]. Based on this different data, this multifunctional miRNA [85] could become a valuable tool for the diagnosis and prognosis of immune activation and chronic inflammation in HIV infection. Moreover, it could be a therapeutic tool regarding its action on SOCS-1 [86,87], and TRIM32 [88].

## 4. Conclusions

We have shown that the abundance, size, and miRNA content of plasma EV of HIV− and HIV+ART donors varies during the day and are more striking in HIV− participants. Results reveal that daily variations in EV abundance and microRNA contents are disrupted in PLWH despite the suppression of HIV replication during ART. This disturbance could explain the mechanism of mRNA and/or protein level changes observed in PLWH and confirm that the timing of biological sample collection matters for analyzing and interpreting study results in comparing HIV uninfected and infected subjects. These different results indicate that EV in plasma are dynamic structures, with their number, size, and miRNA content changing continuously with the circadian rhythm and must be considered in the validation of EV and their content as biomarkers. An improved understanding of EV production and their miRNA content circadian variation can dictate pathological outcomes and how the timing of therapeutic interventions likely determines clinical efficacy in the hope of their future use as therapeutic tools in circadian medicine and circadian immunotherapy.

## 5. Materials and Methods

### 5.1. Population Understudy

PLWH treated with ART (HIV+ART) and having undetectable plasma viral load (≤40 copies per mL) (n = 10) and HIV uninfected subjects (HIV−, n = 10) were recruited at Centre de Recherche du CHU de Montréal (CHUM). The characteristics of the patients are summarized in Table 2. This study received approval from the ethics review boards of Centre de Recherche du CHU de Montréal, Québec, Canada. All subjects were anonymous volunteers and provided written informed consent to participate in the study. Peripheral blood from venipuncture was collected in EDTA-containing tubes, and all participants were sampled at 10:00 and 22:00 on the same day.

### 5.2. Purification of Extracellular Vesicles

Purification of EVs was performed, as we described previously [47]. Briefly, blood obtained by venipuncture with EDTA as an anticoagulant was centrifuged for 10 min at 400× *g* at room temperature to obtain plasma then the plasma was centrifuged again for 10 min at 3000× *g* to obtain platelet-free which is stored at −80 °C until analyze. Platelet-free plasma (250 µL) was treated with proteinase K (1.25 mg/mL, Ambion™, Thermo Fisher Scientific, Waltham, MA, USA) for 10 min at 37 °C. Large EVs were purified by centrifuging proteinase K pretreated plasma for 30 min at 17,000× *g* at room temperature. The resulting supernatant was mixed with 63 µL of ExoQuick-TC™ (SBI via Cedarlane, Burlington, ON, Canada) in an Eppendorf tube and held at room temperature for 30 min. Micro-filtered (0.22 µm pore size membrane) 1× phosphate-buffered saline (PBS) (WISENT Bioproducts, Saint-Jean-Baptiste, QC, Canada) was added to the pellet of large EVs, and centrifuged for 30 min at 17,000× *g*. The supernatant was discarded, and the washed large EV pellet was re-suspended in 250 µL of PBS and kept at 4 °C. Small EVs were obtained from the ExoQuick-TC™ precipitation after centrifuging for 30 min at 1500× *g*. The small EVs pellet were washed by adding PBS and centrifuging at 1500× *g* for 5 min. The washed small EV pellet was re-suspended in 250 µL of PBS by vortex mixing and kept at 4 °C. In our EV purification method, we incubated plasma with proteinase K (1.25 mg/mL) before 17,000× *g* centrifugation. This pre-treatment of plasma with proteinase K is described to critical reduce the amount of non-EV proteins (albumin and the apolipoproteins A-1 and B), which can be co-purify with EV [89,90]. This digestion step destroys proteins and their cargo (mRNA, miRNA), and they were removed with the washing step. In our analysis, we observed that the proteinase K step significantly affects EV size distribution measured by DLS (Figure S4).

### 5.3. Extracellular Vesicles Size Measurement

The size of EV was determined by hydrodynamic radius measurement using a Zetasizer Nano S (Malvern Instruments, Ltd., Malvern, United Kingdom). This technique is based on the light scattering intensity due to the Brownian motion of EVs, characterized by a diffusion constant [91]. The size distribution is obtained from the distribution of diffusion constants using the Einstein–Stokes equation [91]. The measurements were made at a fixed position with an automatic attenuator and at a controlled temperature. For each sample, 100 µL of EV suspension was used, and two measurements were averaged.

### 5.4. EV Flow Cytometry Analysis

Purified EV were stained with lipophilic fluorescent carbocyanine dye DiD (DiIC18(5) solid: 1,1′-dioctadecyl-3,3,3′,3′-tetramethylindodicarbocyanine 4-chlorobenzenesulfonate salt, Invitrogen™, Carlsbad, CA, USA), and carboxyfluoresceine diacetate succinimidyl ester (CFSE) (Invitrogen™, Carlsbad, CA, USA) cell-permeable dye. DiD and CFSE were prediluted respectively 1/100 and 1/500 with 0.22 µm filtered solution of PBS 1X + EDTA (100 mM for final concentration). Then, 40 µL of DiD diluted solution (for final dilution 1/200 (1 ug/mL)) was added to 10 µL of EVs then mixed and incubated for 5 min at 37 °C. After incubation, 50 µL of diluted CFSE was added (for final dilution 1/1000 (1 ug/mL)) and incubated for 15 min under the same condition. Next, 400 µL of filtered PBS 1X +EDTA and 5 µL of 15 µm count beads (Polybead^®^ Microspheres 15.00 µm, Polysciences, Inc., Warrington, PA, USA) were added, vortex mixing, and counting by flow cytometry. A method described previously [47,92] for microparticle analyses was used for EVs analysis by flow cytometry in a FACS Canto II Special Order Research Product cytofluorometer equipped with forward scatter coupled to a photomultiplier tube (FSC-PMT) with the “small particles option” (BD Biosciences, Franklin Lakes, NJ, USA). Gating strategy for the identification and analysis of EV are shown in supplementary data (Figures S6–S8). We further explored the presence of CD63 an endosome-specific tetraspanins marker in large and small EVs and then characterizing the cellular source of circulating large and small EVs in a pooling sample. The purified EVs were stained with lipophilic fluorescent tracer dye DiD or CFSE and next labeled antibodies directed against EVs surface markers: CD4 (17-0048-42), CD8 (11-0089-42), CD14 (11-0149-42) from eBioscience), CD15 (555402 from BD biosciences), CD16 (12-0168-73) and, CD19 (12-0198-42) from eBioscience), CD41 (ab19690 from Abcam), CD63 (IM1165U from Beckman Coulter), and CD235a (11-9987-80 from eBioscience) for flow cytometry analysis (Figure S5).

### 5.5. MicroRNA Quantification

EV suspension (100 µL) was diluted 3:1 in TRIzol LS (Ambion, Life Technologies, Carlsbad, CA, USA) and held at −80 °C. From EVs, total RNA was extracted, mixed with 10 µL of diethylpyrocarbonate water, and quantified (1 µL) using a BioDrop-μLITE kit (Isogen Life Science, Utrecht, The Netherlands). Them, 100 ng of RNA was treated with RNase-free DNase I (Ambion™ Life Technologies) then reverse transcribed using a HiFlex miScript RT II kit according to the manufacturer’s instructions (Qiagen, Hilden, Germany). Mature miR-29a (#MS00003262), miR-29b (#MS00006566), miR-92 (#MS00006594), miR-155 (#MS00031486) and miR-223 (#MS00003871) were detected by quantitative polymerase chain reaction (qRT-PCR) using an miScript primer assay kit and miScript SYBR Green PCR kit (Qiagen). Amplification of mature microRNA as cDNA was performed in a CFX Connect real-time PCR Detection System (BIO-RAD, Hercules, CA, USA) using 40 cycles of 94 °C for 15 s, 55 °C for 30 s, and 72 °C for 30 s. Reaction specificity was confirmed using the melt curve procedure (65–95 °C, 0.5 °C per 5 s) at the end of the amplification protocol according to the manufacturer’s instructions. A standard curve was used for absolute quantification of microRNA.

### 5.6. Procedure for Calculating miRNA Copy Number per Vesicle

This procedure was described previously [47]. Large EVs were separated from 250 µL of plasma, which was then mixed with 63 µL of ExoQuick-TC™. Total RNA was extracted from 100 µL of the small EV and large EV fractions. Small EV yielded about 390 ng of RNA (from the 1.25× dilution due to ExoQuick-TC™) and large EV yielded 321 ng (from 100 µL), which indicates that the plasma contained 4.9 ng/µL associated with small EV and 3.2 ng/µL associated with large EV. Based on RT-qPCR, miRNA was expressed in copies/ng of RNA. EVs counts (obtained by cytofluorometry) were expressed per µL of plasma. Multiplying the miRNA copies/ng of RNA by the RNA per µL of plasma gave miRNA copies/µL. Dividing this result by EVs count per µL gave miRNA copies/EVs in 1 µL of plasma. By multiplying the result by a factor of 1000, we obtain the unit number of miRNA copies per vesicle in 1 mL of plasma.

### 5.7. Statistical Analysis

The time-dependent distribution of the individual observations at 10 a.m. and 10 p.m. was presented as before–after. We used a parametric and non-parametric test for comparisons based on normal distribution tests. A principal component analysis was performed to study the relationship between variables. All correlation coefficients were calculated using Spearman rank correlation and *p* values were 2-tailed. Statistical analyses were performed using GraphPad Prism 8 (GraphPad Inc, San Diego, CA, USA) and RStudio packages (Integrated Development for R. RStudio, PBC, Boston, MA, USA, URL http://www.rstudio.com/ accessed on 3 February 2021). A *p* value ≤ 0.05 was deemed statistically significant.

## Figures and Tables

**Table 1:** Correlation between plasma extracellular vesicles miRNA content and patients’ clinical parameters.

microRNAs	Clinical Parameters		EVs miRNA Content		
		Large EVs		Small EVs	
		a.m.	p.m.	a.m.	p.m.
**miR-29a**	Age	r = −0.76; *p* = 0.0218 *		r = 0.81; *p* = 0.0112 *	
	CD4 T cell count	**r = 0.59; *p* = 0.0806**		r = −0.65; *p* = 0.0490 *	
	CD4/CD8 ratio	r = 0.61; *p* = 0.0873			
**miR-29b**					
	CD4/CD8 ratio	r = 0.84; *p* = 0.0064			
**miR-92**					
	Age		r = −0.79; *p* = 0.0143 *		r = −0.76; *p* = 0.0218 *
	CD4 T cell count		r = −0.90; *p* = 0.0008 *		r = 0.76; *p* = 0.0149 *
	CD4/CD8 ratio	r = 0.84; *p* = 0.0071			
**miR-155**					
	Age	r = −0.71; *p* = 0.0377 *		r = 0.74; *p* = 0.0267 *	
	CD8 T cell count				**r = 0.75; *p* = 0.0255**
	CD4/CD8 ratio	r = 0.61; *p* = 0.0873			
**miR-223**					
	Age	r = −0.78; *p* = 0.0156			
	CD4 T cell count			r = −0.61; *p* = 0.0667 *	
	CD4/CD8 ratio	r = 0.66; *p* = 0.0595		r = −0.62; *p* = 0.0776 *	

* Correlations with miRNA copies per vesicle; Bold correlation: HIV+.

**Table 2:** Clinical data of the study participants.

Characteristics	HIV+ART (n = 10)			HIV− (n = 10)			*p*-Value
Years with HIV (years)	16.10	±	6.03	NA		NA	
Duration of ART (years)	11.80	±	7.38	NA		NA	
Age (years)	52.80	±	4.78	50.00	±	11.77	0.9685
CD4 T-cell (cells/µL)	579	±	156	601	±	199	0.9705
CD8 T-cell (cells/µL)	789	±	214	288	±	151	0.0003
CD4/CD8 ratio	0.74	±	0.24	2.47	±	1.28	<0.0001

Data are presented as the means ± standard deviation. NA, not applicable; ART, antiretroviral therapy.

## Data Availability

The data presented in this study are available in the manuscript main tables and supplementary tables.

## Supplementary Materials

The following are available online at https://www.mdpi.com/article/10.3390/pathogens10050518/s1, Table S1: Summary of vesicle size, count, and microRNA content data. Figure S1: Daily variations of microRNA expression in plasma large and small EVs of HIV− and HIV+ART study participants. Figure S2: Principal component analysis of daily variations in EV mRNA expression in HIV+ART and HIV− study participants. Figure S3: Effect of proteinase K on the recovery of plasma EVs obtained from HIV−1 patients. Figure S4: Impact of proteinase K use in extracellular vesicles size distribution. Figure S5: Large and small EVs quantification and cellular origin. Figure S6: Gating strategy for identifying and analysis of DID and CFSE positive extracellular vesicles in Flow cytometry. Figure S7: Large EVs DID and CFSE positive analysis in Flow cytometry. Figure S8: Small EVs DID and CFSE positive analysis in Flow cytometry.
