# Supplementary material for: Diurnal Variation of Plasma Extracellular Vesicle Is Disrupted in People Living with HIV
Source: Pathogens. 2021 Apr 24;10(5):518. doi: 10.3390/pathogens10050518 (PMC8145918; doi:10.3390/pathogens10050518)
Supplement: Supplementary file 1 [file pathogens-10-00518-s001.zip › pathogens-1144731-supplementary.pdf]

Supplementary files

Table S1: Summary of vesicle size, count, and microRNA content data

Parameters	Control (n=10)				HIV+ART (n=10)			
	Large EVs		Small Evs		Large EVs		Small Evs	
	10:00	22:00	10:00	22:00	10:00	22:00	10:00	22:00
**Size (nm): mean (±SD)**	220.4<br>(38.27)	182.1<br>(48.63)	98.02<br>(21.89)	107.6<br>(24.63)	387.6<br>(104.9)	204.7<br>(41.08)	99.75<br>(16.75)	114.5<br>(31.52)
**Count (EVs / µL of plasma): median (IQR)**	104,082<br>(56,232 - 159,093)	523,974<br>(284,658 - 640,035)	160,710<br>(92,004 - 397,485)	455,334<br>(217,965 - 772,875)	81,048<br>(61,545 - 195,822)	179718<br>(115203 - 613272)	311058<br>(106392 - 722535)	122,496<br>(63,228 - 191,433)
**miR-29a copies/ µg of RNA: median (IQR)**	10,635,497<br>(3,888,211 - 19,591,581)	5,067,5624<br>(32,096,677 - 38,178,7122)	19,782,899<br>(9,600,261 - 79,332,573)	958,221<br>(116,873 - 17,935,395)	2,574,277<br>(940,454 - 22,974,353)	15698165<br>(1150696 - 32558101)	9602666<br>(1663073 - 64790212)	57,543,198<br>(12,554,098 - 179,551,188)
**miR-29a copies per vesicle:median (IQR)**	470<br>(104 - 720)	525<br>(191 - 2,053)	654<br>(189 - 3,149)	20<br>(2 - 268)	209<br>(76 - 527)	213<br>(38 - 859)	165<br>(40 - 877)	1,632<br>(404 - 5,866)
**miR-29b copies/ µg of RNA :median (IQR)**	22238311<br>(12,646,954 - 41,635,533)	66,993,839<br>(49,488,489 - 412,721,371	26,911,852<br>(19,810,050 - 916,311,330)	13,278,259<br>(1,100,342 - 33,405,593)	6,179,229<br>(1,001,982 - 32,304,881)	18,155,347<br>(3,251,550 - 41,497,878)	12,549,930<br>(2,445,643 - 39,880,979)	41,576,077<br>(18,804,677 - 90,643,433)
**miR-29 copies per vesicle :median (IQR)**	921<br>(373 - 1420)	555<br>(288 - 1991)	990<br>(313 - 29,778)	115<br>(12 - 880)	415<br>(119 - 753)	527<br>(100 - 840)	169<br>(22 - 636)	1,506<br>(709 - 3,587)
**miR-92 copies/ µg of RNA :median (IQR)**	11558186<br>(5929533 - 33608308)	35819212<br>(20910021 - 88983839)	22,796,978<br>(12,851,309 - 31,331,574)	4,550,714<br>(1,356,622 - 15,616,976)	5,807,012<br>(1,061,745 - 22,912,048)	13,444,335<br>(2,444,418 - 31,914,685)	5,040,032<br>(2,566,729 - 34,389,453)	30,280,865<br>(11,139,507 - 52,302,600)
**miR-92 copies per vesicle :median (IQR)**	556<br>(149 - 1160)	232<br>(175 - 592)	488<br>(157 - 1,137)	65<br>(20 - 238)	372<br>(93 - 577)	259<br>(62 - 714)	154<br>(21 - 262)	1,308<br>(576 - 1,995)
**miR-155 copies/ µg of RNA :median (IQR)**	21602<br>(16750 - 41367)	61,495<br>(50,238 - 2,439,734)	4,302<br>(1,316 - 9,096)	2,281<br>(914 - 97,88)	10153<br>(4,332 - 44,307)	23,905<br>(7,587 - 79,825)	2,827<br>(1,379 - 4,389)	9401<br>(4,322 - 1,1791)
**miR-155 copies per vesicle :median (IQR)**	0.80<br>(0.34 - 2)	0.58<br>(0.29 - 13)	0.08<br>(0.02 - 0.36)	0.02<br>(0.01 - 0.14)	0.74<br>(0.32 - 1)	0.64<br>(0.24 - 2)	0.05<br>(0.004 - 0.10)	0.29<br>(0.19 - 1.47)
**miR-223 copies/ µg of RNA :median (IQR)**	54524349<br>(21499006 - 83726387)	577,505,505<br>(396,803,676 - 941,484,115)	105,510,084<br>(27,838,867 - 168,313,129)	2,602,607<br>(175,721 - 46,421,623)	925,5782<br>(3,212,461 - 88,988,310)	484,66,146<br>(7,261,969 - 82,514,910)	12,234,159<br>(3,359,861 - 90,144,545)	44,570,585<br>(1,558,7534 - 99,801,099)
**miR-223 copies per vesicle :median (IQR)**	1707<br>(343 - 3884)	4254<br>(2,295 - 6,530)	1,996<br>(1,010 - 4,328)	48<br>(4 - 745)	692<br>(235 - 2,214)	913<br>(186 - 2052)	181<br>(51 - 1181)	1,601<br>(685 - 3,873)

Supplementary files

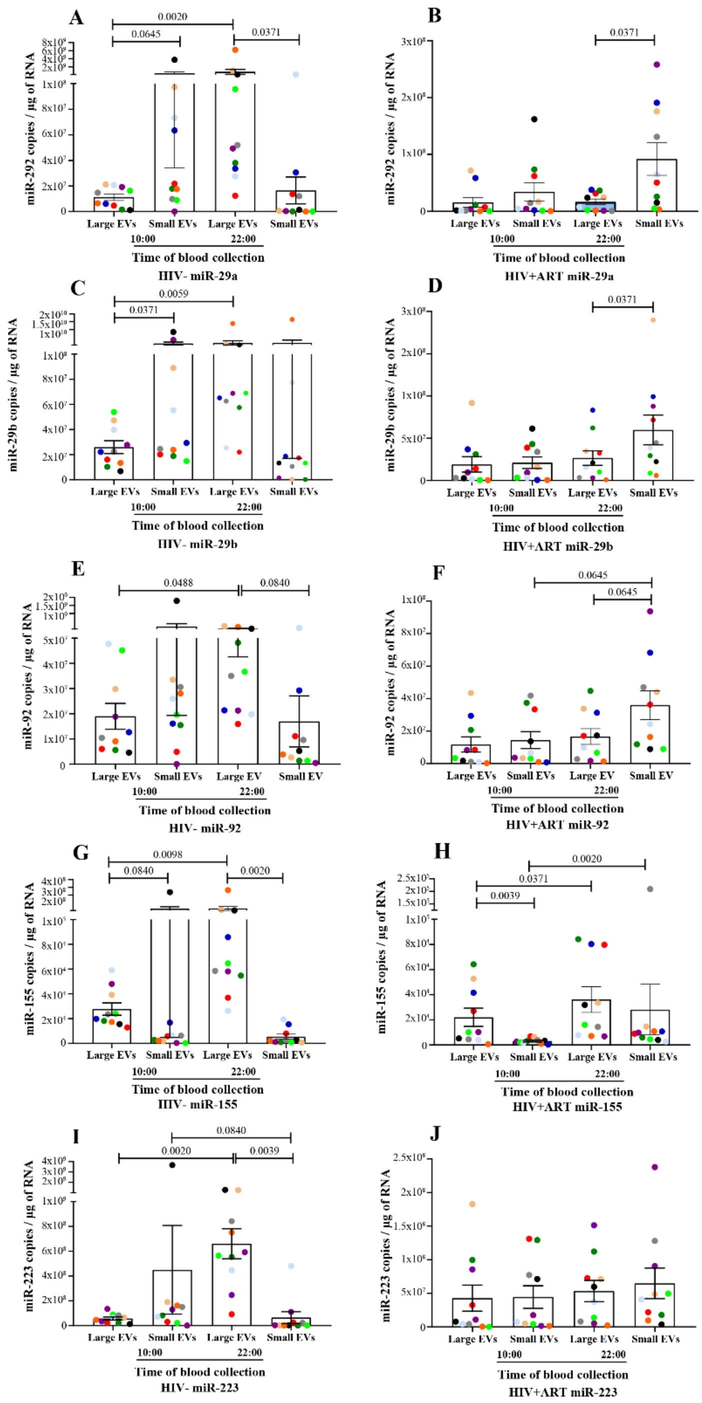

Figure S1: Daily variations of microRNA expression in plasma large and small EVs of HIV- and HIV+ART study participants.

Total RNA was extracted from large and small EVs purified from platelets-free plasma of uninfected (HIV-, n = 10) and HIV infected ART-treated subjects (HIV+ART, n = 10) sampled at 10:00 and 22:00 the same day. Matures miRNAs were reverse transcribed and quantified as described in the Materials and Methods section in large and small. (A and B) miRNA-29a, (C and D) miR-29b, (E and F) miR-92, (G and H) miR-155 and (I and J) miR-223 expression level in HIV- and HIV+ART. Wilcoxon matched-pairs signed rank test was used for statistical analysis.

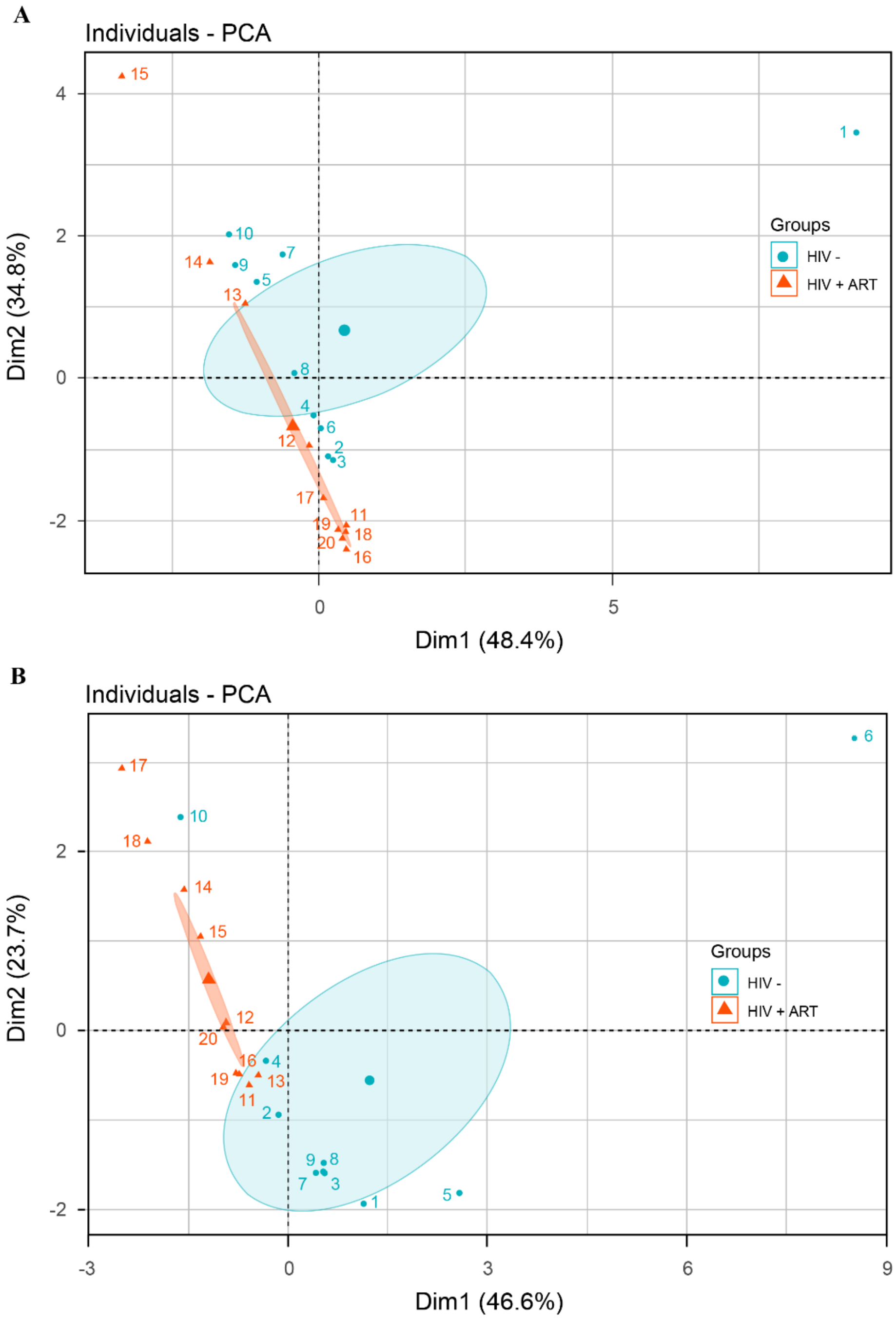

Figure S2: Principal component analysis of daily variations in EV mRNA expression in HIV+ART and HIV- study participants. Correlation graph of 10 control subjects (individuals 1-10) and 10 HIV infected subjects (individuals 11-20) according to their large and small EVs miRNA content at 10:00 (A) and 22:00 (B).

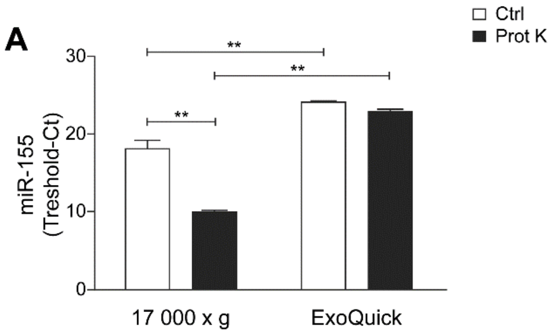

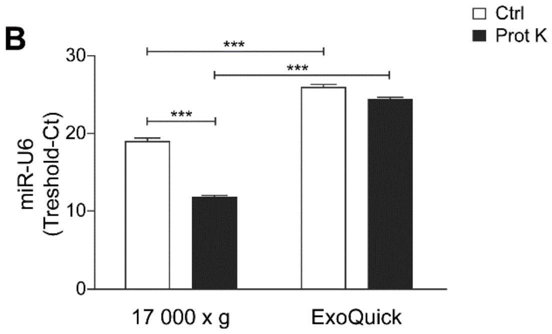

Figure S3: Effect of proteinase K on the recovery of plasma EVs obtained from HIV-1 patients. Plasma was treated with proteinase K before centrifugation at 17,000 x g to obtain large EV (microvesicles) followed by ExoQuickTM precipitation of EVs to obtain small (EV) according to the procedure specified by the manufacturer. RT-PCR was used to amplify microRNA: miR-155 (A) or U6 (B). These results confirm the efficiency of proteinase K treatment.

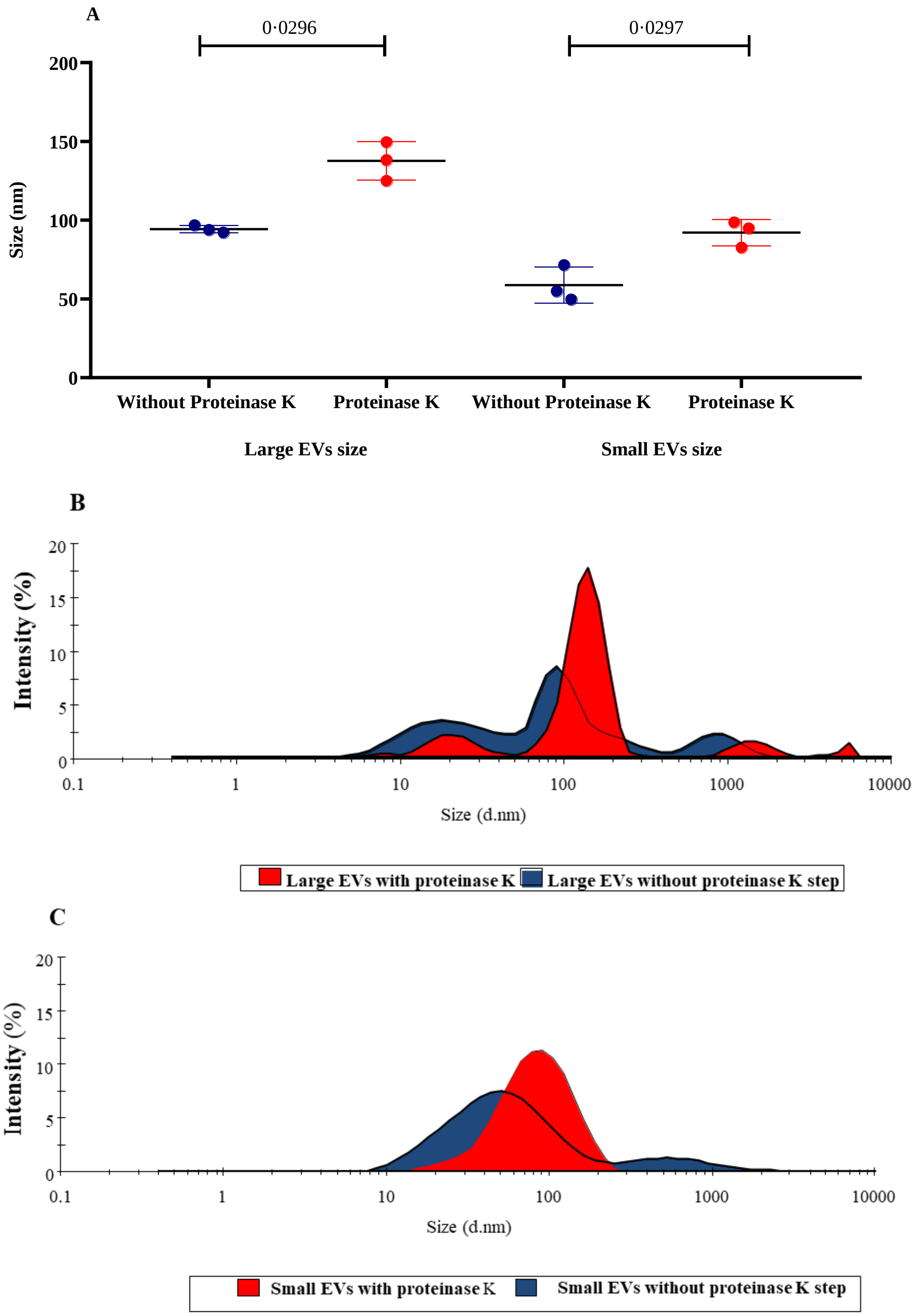

Figure S4: Impact of proteinase K use in extracellular vesicles size distribution. Large and small EVs were purified from the plasma of 3 HIV uninfected patients with and without plasma proteinase K pre-treatment step. Panel A presents large and small EVs size with and without proteinase K step. Panel B and C show large and small EVs size distribution without proteinase K step in blue and with proteinase K step in red based on DLS measurement. (EVs: extracellular vesicles). A paired t test was used for statistical analysis.

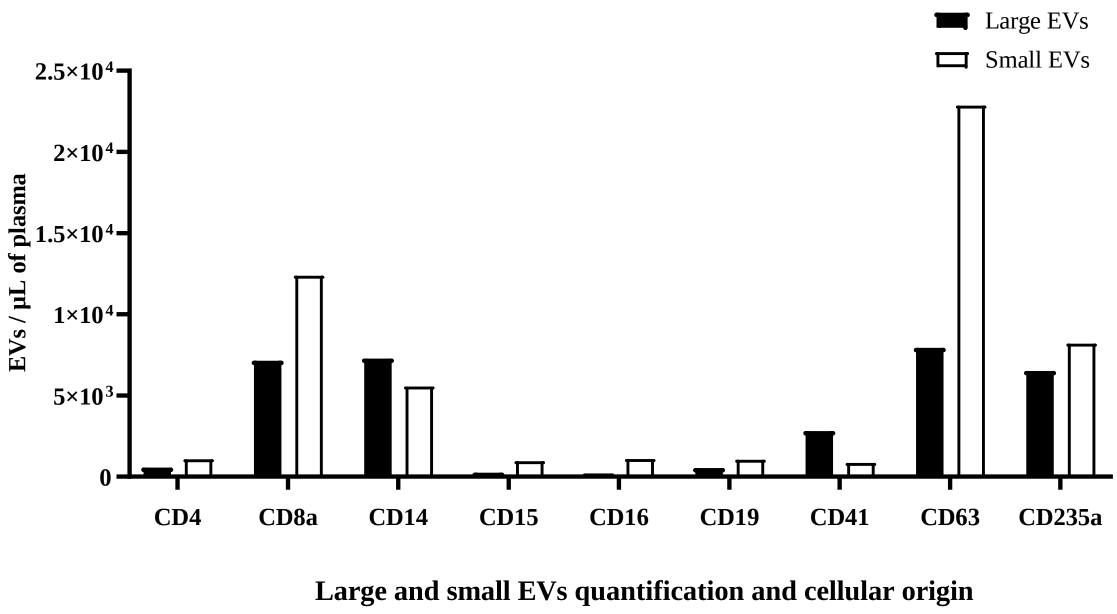

Figure S5: Large and small EVs quantification and cellular origin. Large and small EVs were purified from pooling plasma of 300 patients (240 HIV infected and 60 HIV (uninfected) without plasma proteinase K pre-treatment step. The purified EVs were stained with lipophilic fluorescent tracer dye DiD or CFSE and then marked with antibodies directed against receptors CD4, CD8, CD14, CD15, CD16, CD19, CD41, CD63, and CD235a for flow cytometry analysis.

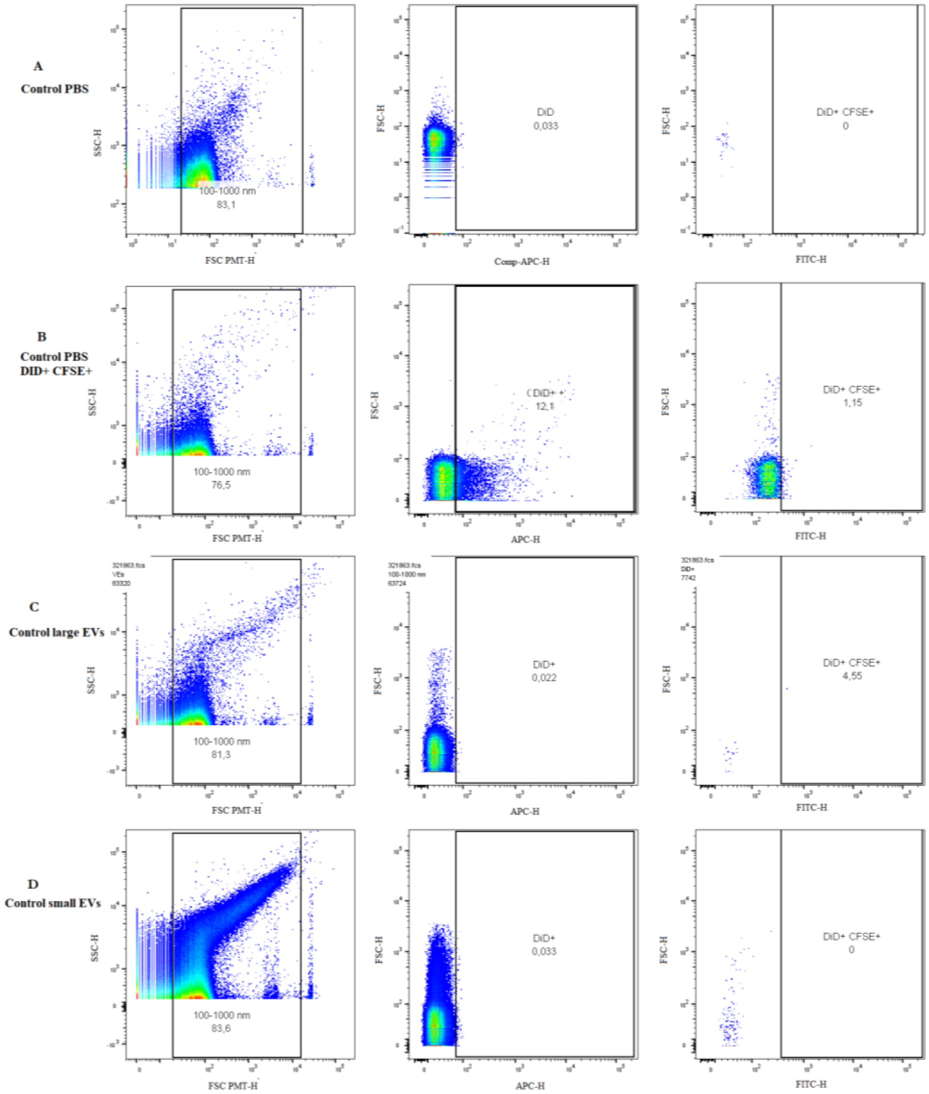

Figure S6: Gating strategy for identifying and analysis of DID and CFSE positive extracellular vesicles in Flow cytometry. Large and small EVs were purified from HIV infected ART-treated subjects (HIV+ART, n = 10) and uninfected (HIV-, n = 10) participants proteinase K pretreated plasma sampled at 10:00 and 22:00 on the same days. A gate representing the size range of interest (100 to 1000 nm) was set according to known diameters of standard beads, and positive fluorescent events were used to detect EV and portrayed in an SSC/FSC-PMT graph. Total DID+ events are detected in the 100 to 1000 nm gate and the quantity of EV CFSE+ is determined in the DID+ EVs gate. **A)** Buffer only (0.22mm filtered pore size membrane PBS) was used to determine the background signal. Then lipophilic fluorescent carbocyanine dye DiD and the CellTrace CFSE (carboxyfluorescein diacetate succinimidyl ester (CFDA-SE, hereinafter CFSE) were incubated in PBS EV free and acquired to determine any particle signal arising from both reagents. (**A**) Shows the scatter intensity of buffer alone (without EV). (**B**) Similar results with the addition of the lipophilic dye carbocyanine DiD and CFSE to buffer only (without EV). (**C and D**) shows an example of a gating strategy applied to large and small EVs without staining under the same acquisition parameters applied in for PBS (A and B).

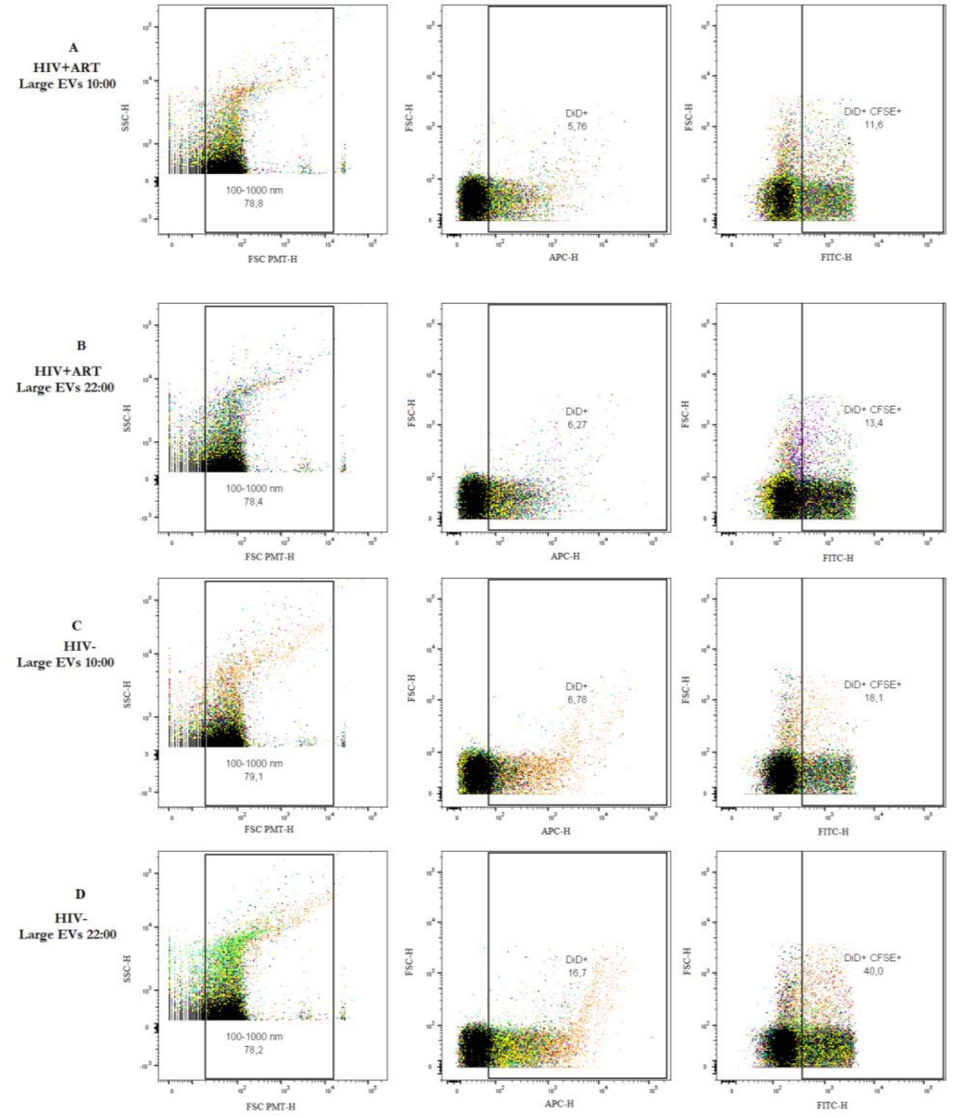

Figure S7: Large EVs DID and CFSE positive analysis in Flow cytometry. Large EVs were purified from HIV infected ART-treated subjects (HIV+ART, n = 10) and uninfected (HIV-, n = 10) participants proteinase K pretreated plasma sampled at 10:00 and 22:00 on the same day. EVs were incubated with lipophilic dye carbocyanine DiD and carboxyfluorescein diacetate succinimidyl ester (CFSE) as described in the methods section. Otherwise, the same acquisition parameters as in control (Fig S) were applied. The estimated size range of 100 to 1000 nm DID and CFSE positive events were identified as intact EV. (**A and B**) Present individual pooling result of large EVs from HIV+ART (n=10) respectively in 10:00 and 22:00 sample. (**C and D**) Present individual pooling result of large EVs from HIV- (n=10) respectively in 10:00 and 22:00 sample.

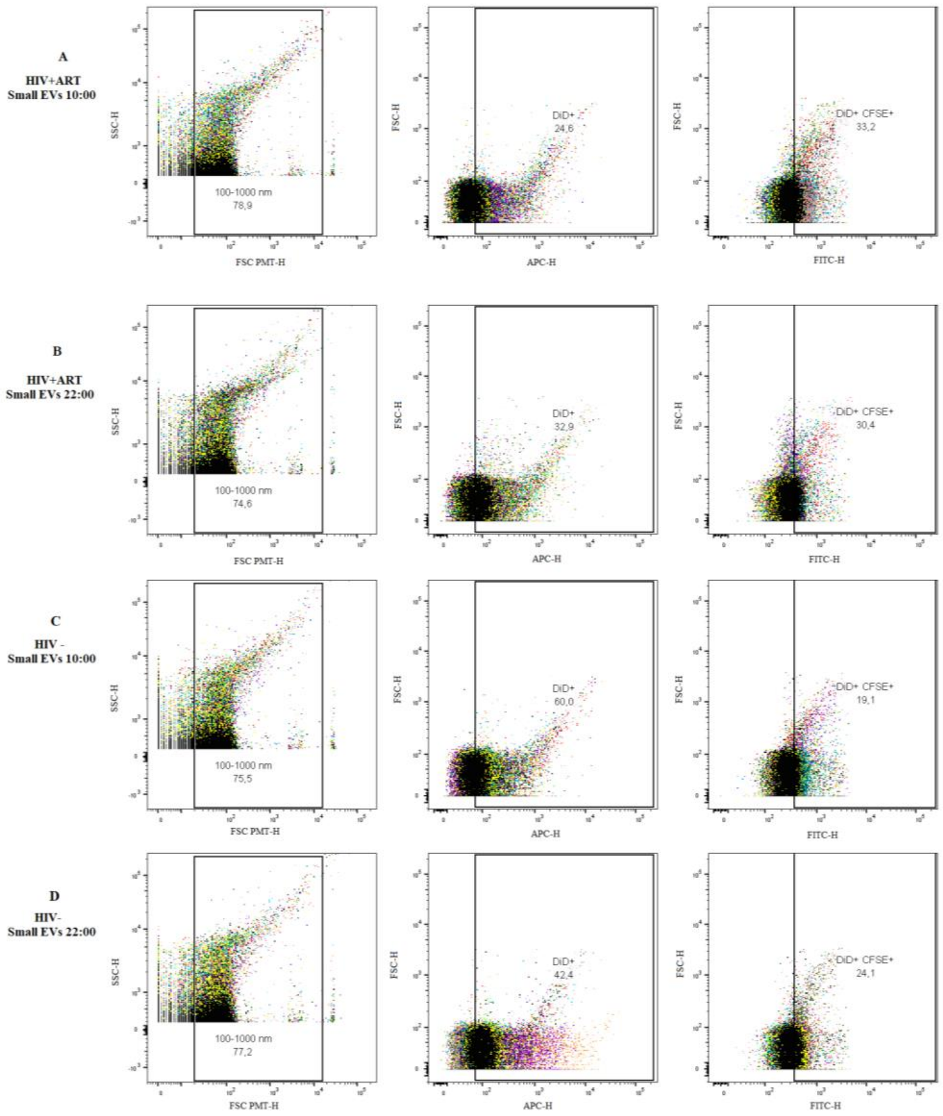

Figure S8: Small EVs DID and CFSE positive analysis in Flow cytometry. Small EVs were purified from HIV infected ART-treated subjects (HIV+ART, n = 10) and uninfected (HIV-, n = 10) participants proteinase K pretreated plasma sampled at 10:00 and 22:00 on the same days. EVs were incubated with lipophilic dye carbocyanine DiD and carboxyfluorescein diacetate succinimidyl ester (CFSE) as described in the methods section. Otherwise, the same acquisition parameters as in control (fig S) were applied. The estimated size range of 100 to 1000 nm DID and CFSE positive events were identified as intact EV. (**A and B**) Present individual pooling result of small EVs from HIV+ART (n=10) respectively in 10:00 and 22:00 sample. (**C and D**) Present individual pooling result of small EVs from HIV- (n=10) respectively in 10:00 and 22:00 sample.
